# Os odontoideum: database analysis of 260 patients regarding etiology, associated abnormalities, and literature review

**DOI:** 10.3389/fsurg.2023.1291056

**Published:** 2023-12-05

**Authors:** Arnold H. Menezes

**Affiliations:** Neurosurgery & Pediatrics, University of Iowa Hospitals & Clinics, University of Iowa Stead Family Children’s Hospital, Iowa City, IA, United States

**Keywords:** Chiari I malformation, craniovertebral junction, Down syndrome, os odontoideum, spondyloepiphyseal dysplasia

## Abstract

**Introduction:**

Since the first description of os odontoideum in 1886, its origin has been debated. Numerous case series and reports show both a possible congenital origin and origin from the secondary to craniovertebral junction (CVJ) trauma. We conducted a detailed analysis of 260 surgically treated cases to document the initial symptoms, age groups, radiographic findings, and associated abnormalities, aiming to enhance the confirmation of the etiology. A literature search (1970–2022) was performed to correlate our findings.

**Methods and materials:**

A total of 260 patients underwent surgical management of a referral database of 520 cases (1978–2022). All patients were examined by plain radiography and myelotomography as needed until 1984, and since then, CT and MRI have been employed. History of early childhood (aged below 6 years) CVJ trauma was investigated, including obtaining emergency department's initial radiographs from the referral and subsequent follow-up. Associated radiographic and systemic abnormalities were noted, and the atlas development was followed.

**Results:**

The age of the patients ranged from 4 to 68 years, mostly between 10 and 20 years. There were 176 males and 86 females. Orthotopic os odontoideum was identified in 24 patients, and 236 patients had dystopic os odontoideum. Associated abnormalities were found in 94 of 260 patients, with 73 exhibiting syndromic abnormalities and 21 having Chiari I malformation. Two sets of twins had spondyloepiphyseal dysplasia. Of 260 patients, 156 experienced early childhood trauma /. Among these, 54 initially presented with normal radiographs but later demonstrated anterior atlas hypertrophy. In addition, a smaller posterior C1 arch was observed, leading to the development of os odontoideum. Two children had initial CVJ trauma as documented by MRI, with subsequent classical findings of os odontoideum and atlas changes. Syndromic patients had an earlier presentation. The literature reviewed confirms the multifactorial etiology.

**Conclusions:**

The early presentation and associated abnormalities (such as Down syndrome, Klippel–Feil syndrome, Chiari I malformation, spondyloepiphyseal dysplasia, Morquio syndrome, and others) along with case reports documenting familial, hereditary, and twin presentations strongly support a congenital origin. Likewise, surgical complications are more prevalent in syndromic patients (40%) compared to 15% in other cases, as reported in the literature. The documentation of normal odontoid in early childhood trauma cases followed by the later development of os odontoideum provides evidence supporting trauma as an etiological factor. This process also involves vascular changes in both the atlas and the formation of os odontoideum. Associated abnormalities exhibit an earlier presentation and are only seen in cases with a non-traumatic origin.

## Introduction

Radiographically, os odontoideum is described as an independent ossicle located posteriorly to the hypertrophic anterior atlas arch, featuring cortical margins, distinct from a hypoplastic dens (1). The anterior arch of the atlas appears enlarged, accompanied by the presence of a small posterior arch. This differs from a recent odontoid fracture. There are two varieties of os odontoideum: the first, termed orthotopic, is positioned behind the anterior arch of the atlas in the normal location where the odontoid process would be (2). The second variety involves cranial migration of the os odontoideum, moving in conjunction with the clivus, and is termed dystopic. The gap beneath the os odontoideum can lead to the incompetence of the cruciate ligament and subsequent craniocervical instability. Even though the volume of literature on os odontoideum has been expanding, questions about its etiology remain a topic of debate, despite the occurrence of congenital, familial, and associated abnormalities (3–14). Moreover, early childhood trauma at the craniocervical junction is one of the leading causes of the traumatic formation of os odontoideum (3, 4, 9). We have conducted an analysis of a large database focusing on both the congenital and traumatic origins of os odontoideum, including associated abnormalities. A literature review spanning from 1970 to 2022 was conducted to validate our findings. The exploration of embryology and blood supply plays a major role in enhancing our understanding of the issue (15–18).

## Methods and materials

The referral database for craniovertebral junction (CVJ) abnormalities was initiated in 1978 (19). Out of a referral base of 520 cases from 1978 to 2022, surgical management was conducted on 260 patients with os odontoideum(). All inpatient and outpatient records were retrospectively reviewed. The following data and variables were recorded: patient age, sex, age of symptomatic presentation, anatomical classification of the os odontoideum, and associated craniocervical junction and systemic abnormalities. All patients were examined by plain radiography and myelotomography as needed until 1984, and since then, CT and MRI have been employed (2). A history of early childhood craniovertebral junction trauma (less than 6 years) was investigated, which included obtaining initial radiographs from emergency departments from the national referral and subsequent follow-up (9). Associated radiographic and systemic abnormalities were noted, and the abnormal atlas development was followed up.

Prior to 1984, all patients underwent plain cervical spine radiography and pluridirectional tomography in the flexed, extended, and lateral bending positions. This was further supplemented with CT imaging, CT myelography, and, at times, vertebral angiography. After 1984, CT myelography was replaced with MRI in the flexed and extended positions, along with CT angiography. 3D CT imaging of the CVJ has been performed in all patients since 1990. Particular attention has been given to facet joint abnormalities in both the forward review and retrospective analysis (20). This manuscript does not delve into the specifics of surgical management.

## Results

Patients ranged in age from 4 to 68 years. The age group with the highest number of patients was 10–20 years (Table 1). The youngest symptomatic patient in our study was 4 years old, with os odontoideum development. Children with associated congenital abnormalities presented at an earlier stage (Table 2), although they might have become symptomatic a few years prior. There were 176 males and 86 females in the study.

**Table 1:** Age at presentation.

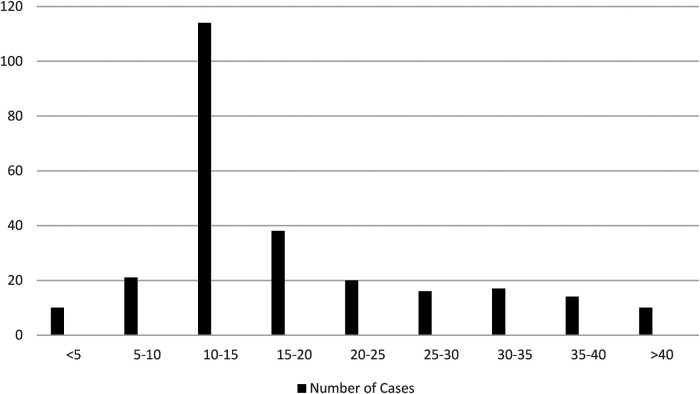	
Age (years)	Number of cases
<5	10
5–10	21
10–15	114
15–20	38
20–25	20
25–30	16
30–35	17
35–40	14
>40	10

**Table 2:** Associated conditions with os odontoideum (260 patients).

Pathology	#	Age at presentation (years)	Orthotopic	Dystopic
Klippel–Feil syndrome^a^	26	All ages	9	17
Chiari I malformation^a^	20	All ages	0	20
Down syndrome	11	2–5	7	4
Atlas assimilation^a^	8	2–5	5	3
Bipartite atlas arches	6	4–6	0	6
Spondyloepiphyseal dysplasia	6	6–10	4	2
Morquio syndrome	4	5–10	0	4
Craniofacial dysostosis^a^	4	8–10	4	0
Spondylometaphyseal dysplasia	2	<5	2	0
Larsen syndrome	2	<5	2	0
Dandy–Walker malformation	2	<5	2	0
Charge syndrome	1	5	1	0
Achondroplasia	1	10	0	1
Chondrodysplasia ossificans	1	6	1	0
Total	94			

^a^ Had combination or other abnormalities.

### Radiographic findings

Applying a radiographic classification of os odontoideum, the authors identified 24 cases of orthotopic os odontoideum and 236 cases of dystopic os odontoideum. Out of 260 patients, 94 (36%) had associated abnormalities, with 73 exhibiting syndromic abnormalities and 21 having Chiari I malformation. In this congenital variety, a combination of segmentation failure and Chiari abnormality was observed. The associated abnormalities were evidently more prevalent in the pediatric cohort than in adults.

Within the large database of 520 patients, 64 were incidentally identified as having os odontoideum but did not receive initial treatment. Of the 64 patients, 28 exhibited later instability, with a mean of 4 years after diagnosis; among these, 8 were children.

We were unable to identify any variables, such as osseous abnormalities at the atlantoaxial facet joints, that could prevent reduction (20). However, this observation could reflect the characteristics of the patient population.

Early in the series, we encountered two sets of twins with a family history of spondyloepiphyseal dysplasia, both presenting with an unstable os odontoideum (likely ossiculum terminale). Of interest to note is that four children who had previously undergone craniofacial reconstruction for craniofacial dysostosis later showed reducible dystopic os odontoideum.

A history of early childhood craniocervical junction trauma (less than 6 years of age) was investigated, which included obtaining initial radiographs from emergency departments from the national referral and subsequent follow-up. Of the 260 patients, 156 had a previous history of early childhood trauma. We obtained cervical spine documentation of a normal odontoid process at the initial injury in 54 of the 156 patients who subsequently developed os odontoideum. Recently, two patients were documented with craniocervical ligamentous injuries on MRI. Attention was paid to the development of the anterior atlas arch, especially focusing on the anterior tubercle with hypertrophy in post-traumatic cases including gradual shrinkage of the posterior atlas arch. This is further reviewed in the Discussion section of this manuscript. It is related to both the blood supply and embryology.

## Illustrative cases

### Case 1

**Figure 1:**
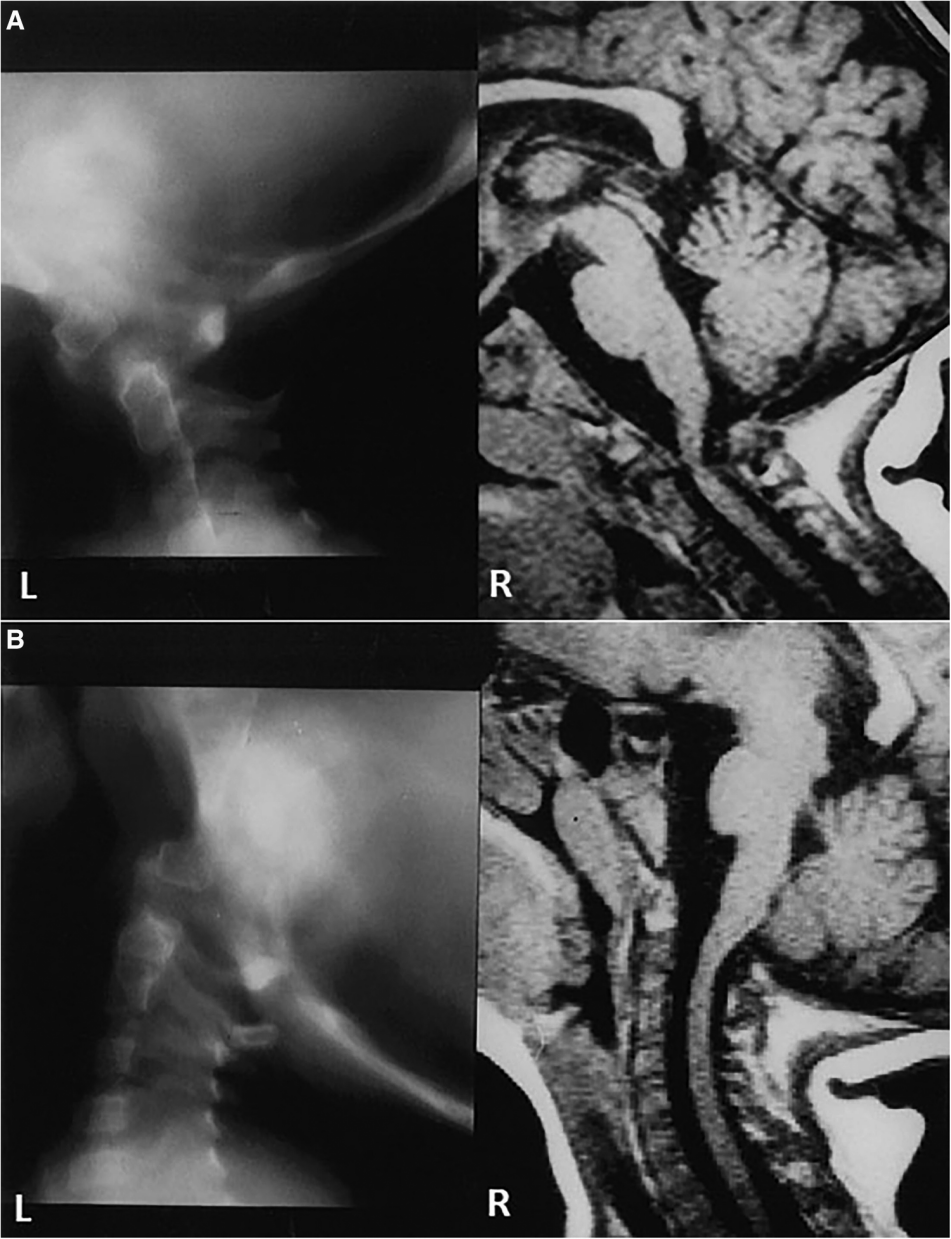
(**A**) Composite of midline pluridirectional CVJ tomogram (L) and midsagittal T2-W CVJ MRI in flexion (R). The posterior C1 arch moves forward, and the os odontoideum is seen anteriorly. (**B**) Same patient as in (**A**): extension studies.

In 1989, this 4.5-year-old girl presented with weakness in the arms, numbness in the hands, and a peculiar gait. She had a dystopic os odontoideum with abnormal motion dynamics. The anterior and posterior arches of the atlas appeared to be of normal caliber, but forward motion of the posterior atlas arch in flexion was observed, leading to compression at the cervicomedullary junction, as seen in both pluridirectional tomography and flexion MRI scans (Figure 1A). This was corrected in extension (Figure 1B). She underwent intraoperative crown halo traction and a dorsal occipitocervical fusion procedure using a rib graft. This demonstrates the normal thickness of the anterior and posterior atlas arches, both of congenital origin, accompanied by abnormal motion dynamics.

### Case 2

**Figure 2:**
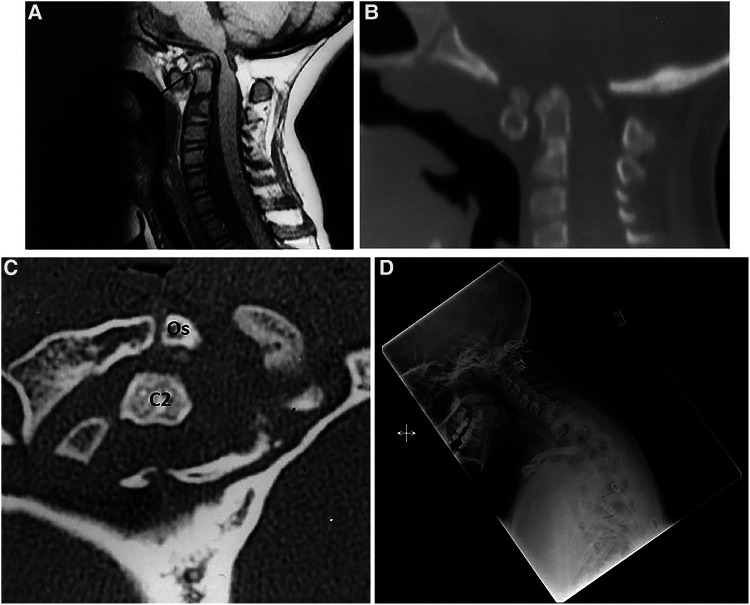
(**A**) Midsagittal T1-W MRI of CVJ in a 6-year-old child. Note the cervicomedullary compression by the superior C2 and the dorsal C1 arch. There is an os odontoideum. The arrow demonstrates the cruciate ligament. (**B**) 2D CT of CVJ of the patient in (**A**). (**C**) Axial CT at C1. The anterior arch of C1 is bifid with the os odontoideum in the bipartite space. (**D**) Lateral cervical radiographs obtained 11 years later. Note the normal alignment and dorsal occipitocervical fusion.

In 2001, a 6-year-old girl presented with short stature and progressive quadriparesis. A combination of MRI and CT revealed a dislocation at the C1–C2 level, along with os odontoideum and a cruciate ligament over the C2 vertebral body (as indicated by an arrow and confirmed during transoral surgery). There was an hourglass compression of the cervicomedullary junction (Figure 2A). The CT scan demonstrated bifid anterior and posterior arches of C1, with the os odontoideum occupying the space of the diastatic anterior arch of C1 (Figures 2B,C). She underwent an anterior transoral resection of the os odontoideum and the superior aspect of the axis body. She also had a C1 laminectomy with dorsal occiput–C3 fusion using a rib graft (Figure 2D). She achieved a complete neurological recovery.

### Case 3

**Figure 3:**
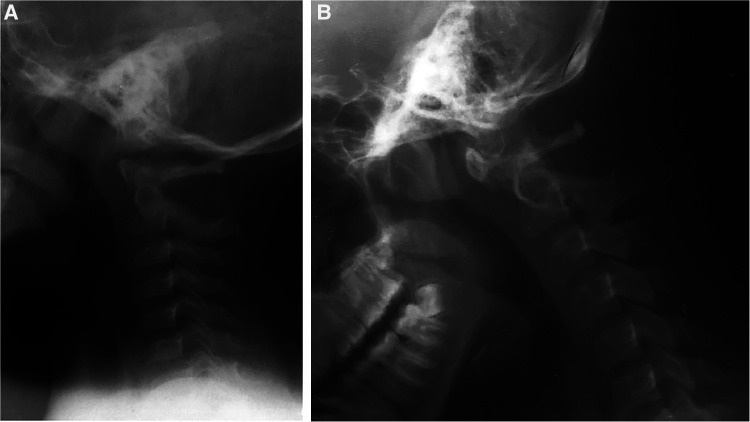
(**A**) Lateral cervical spine radiograph after motor vehicle accident of a 4-year-old girl, in 1986. Note the widening of C1–C2 interspinous space and the intact odontoid process. (**B**) Lateral cervical spine X-ray obtained 10 years later [patient in (**A**)]. There is a C1–C2 dislocation and os odontoideum.

In 1986, a 4-year-old girl was involved in a motor vehicle accident. The lateral cervical spine radiographs from a local emergency department admission showed an intact odontoid process with widening of the posterior atlantoaxial space and straightening of the cervical spine (Figure 3A). She did well. At the age of 14 years, she presented to us with significant neck pain and paresthesias in her hands. Cervical spine radiographs demonstrated a dystopic os odontoideum (Figure 3B). She underwent a dorsal occipitocervical fusion. This demonstrates a post-traumatic development of os odontoideum. The anterior atlas arch and the anterior tubercle are hypertrophied, while the posterior arch is small and atrophic.

### Case 4

**Figure 4:**
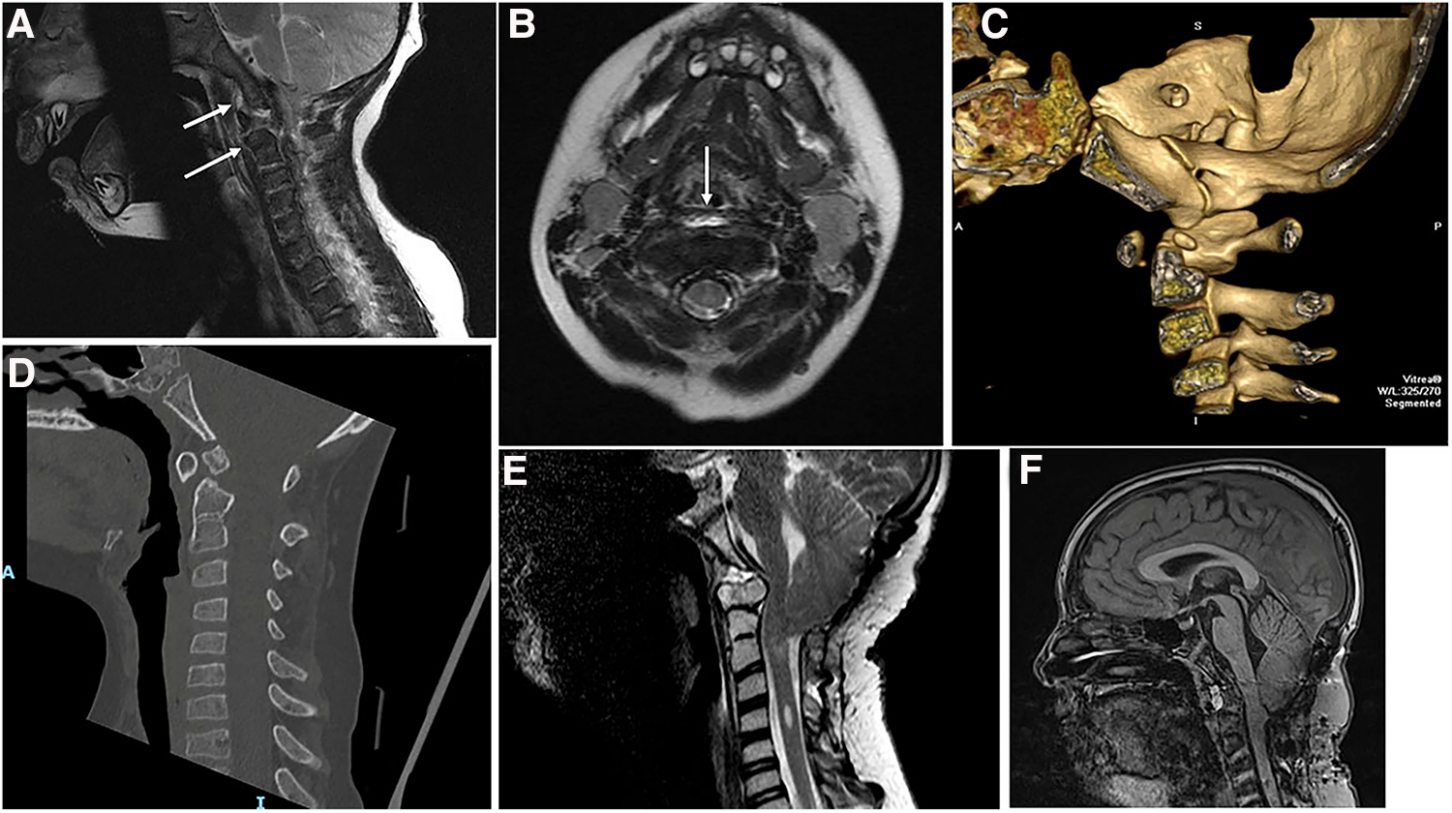
(**A**) Midsagittal T2-W MRI of CVJ of an 18-month-old boy. There is a prevertebral bright signal in front of C1–C2 vertebral bodies, (arrows) Chiari I abnormality, and a widened cervicomedullary junction. (**B**) Axial T2-W MRI through C1of the same patient as in (**A**). Note the prevertebral signal abnormality (arrow). (**C**) 3D CT of CVJ in midsagittal reconstruction made at the age of 2.5 years. A small ossicle is seen above the odontoid. (**D**) 2D CT of CVJ reveals an os odontoideum at the age of 10 years. (**E**) Midsagittal T2-W MRI of CVJ after posterior fossa decompression and dorsal occipitocervical fusion. There is ventral compression by the os odontoideum. (**F**) Midsagittal T2-W MRI of CVJ done after transoral resection of the os odontoideum.

In 2006, an 18-month-old boy was referred to our facility after falling out of a crib; due to an inability to sit and poor hand movement. He exhibited ecchymosis on his forehead, prompting immediate magnetic resonance imaging with a cervical collar for stabilization. The cervicomedullary junction was enlarged, and there was 8-mm tonsillar herniation, consistent with Chiari I abnormality (Figures 4A,B). On T2-W MRI, there was an area of linear brightness, indicating elevation of the anterior longitudinal ligament in front of C1, C2, and C3 vertebral bodies. A small odontoid process was identified (Figures 4A,B). The assessment suggested craniocervical ligamentous injury, cervicomedullary contusion, and Chiari I abnormality. Significant neurological improvement was observed with the administration of dexamethasone and the use of a cervical brace. Lateral cervical spine radiographs at 2 months showed incomplete development of the anterior and posterior arches of the atlas with normal alignment. Repeat MRI showed soft tissue crowning of the odontoid process and cerebellar tonsillar ectopia. The prevertebral swelling had receded. We investigated the alar and cruciate ligaments, as well as the tectorial membrane, and found them to be intact. A year following his injury, a CT scan of the neck showed irregular ossification of the axis body, a hypoplastic dens, and a small odontoid process (Figure 4C). Seven years later, at age 9, a lateral cervical spine radiograph was performed elsewhere after a school altercation, revealing a dystopic os odontoideum. He subsequently presented to us again with complaints of headaches, difficulty swallowing, bladder incontinence, numbness in his hands, and a tendency to drop objects (Figure 4D). Magnetic resonance imaging showed a Chiari malformation, with the tonsils descending to 20 mm below the plane of the foramen magnum. Gross atlantoaxial instability was observed on cervical dynamic radiographs. He underwent a posterior fossa procedure for Chiari I abnormality and dorsal occipitocervical fusion (Figure 4E). He later presented with progressive Lhermitte's phenomenon and achieved full recovery following transoral decompression of the cervicomedullary junction (Figure 4F). A bony spicule from the posterior surface of the os odontoideum had penetrated the dura.

This is MRI documentation of craniocervical ligamentous injury with sequential formation of os odontoideum.

## Discussion

### Embryology and vascular supply of the bony architecture of the craniovertebral junction

The relevant embryology is summarized to provide an understanding of the etiology of os odontoideum (2, 15, 18). At 4 weeks gestation, there are 42 somites, of which four are occipital and eight are cervical. Each somite divides into an outer dermatome, an inner myotome, and a medial sclerotome. The sclerotomes are located ventromedially and will form the vertebral bodies. The first four sclerotomes will fuse to form the occipital bone and the posterior portions of the foramen magnum. There are simultaneous vascularization and differentiation of the ganglia and vascular tissues. The fourth sclerotome serves as the foundation for understanding the craniocervical junction and is termed proatlas.

**Figure 5:**
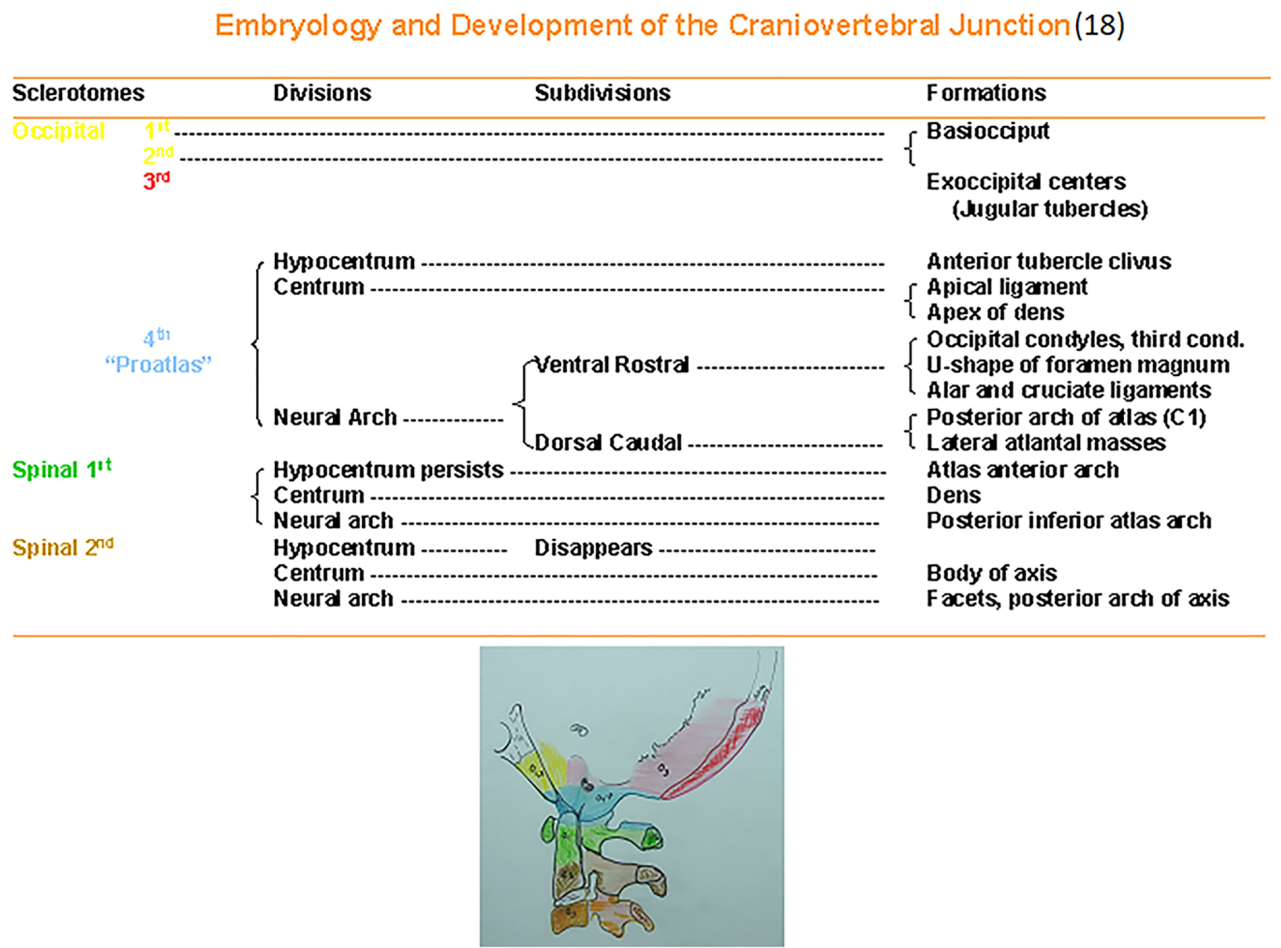
Embryology and development of the craniovertebral junction (18).

**Figure 6:**
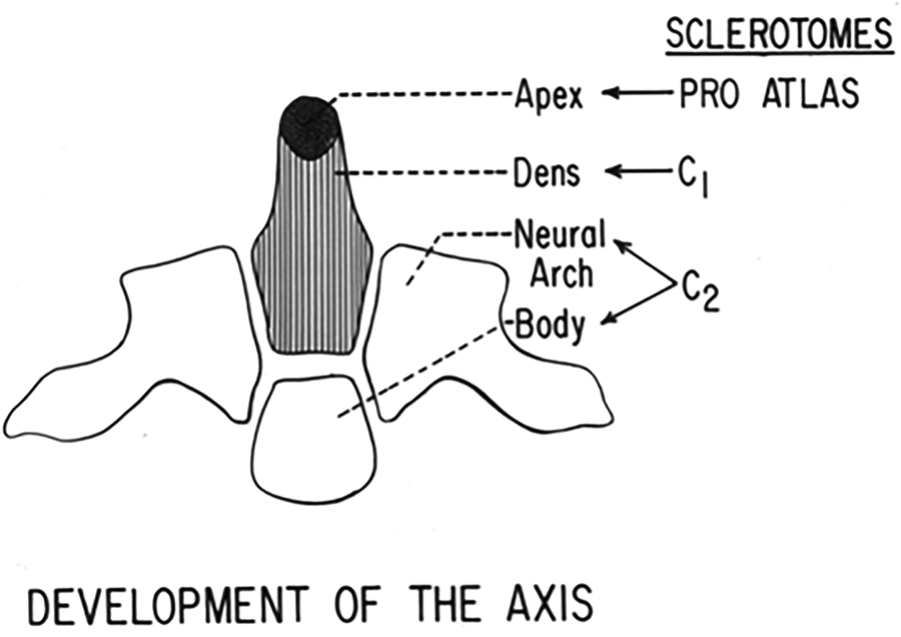
Development of the axis vertebra.

Figure 5 identifies the embryology and development of the craniocervical junction and provides ease of reference. The proatlas divides into a ventral rostral segment and a corresponding dorsal caudal portion. This latter forms the posterior arch and the lateral masses of the atlas. The ventral component of the proatlas forms the anterior U-shaped margin of the foramen magnum and the occipital condyles. A portion of the ventral component of the proatlas detaches and integrates with the pleural centrum of the atlas vertebra, which develops as a dens. The core of the atlas centrum is metamorphosized into the apical ligament of the dens. The atlas, the check ligaments, and the transverse ligament of the atlas are derived from the proatlas as unossified tissue. The dens component of the odontoid process actually represents the atlas centrum. At birth, the odontoid is separated from the body of the axis by a cartilaginous band, representing a vestigial disc referred to as neurocentral synchondrosis (Figure 6). This lies below the level of the superior articular facets of the axis and does not represent the anatomical basis of the dens. Neurocentral synchondrosis is present in nearly all children by the age of 3 years and is absent in most by the age of 6–8 years. At birth, a recognizable odontoid process should be which has not yet fused to the base of the axis. The tip of the odontoid process is not ossified and is represented by a separate ossification center (from proatlas), which is usually seen at age 3 years and fuses with the body of the dens. A second spinal sclerotome contributes to the caudal portion of the axis body and the arches.

The axis vertebra is biomechanically adapted to encourage motion while preserving stability (21). The complex anatomy of the craniovertebral articulation provides stability reinforced by the ligaments and membranes that contribute to the stability of the occipital–atlantoaxial complex.

**Figure 7:**
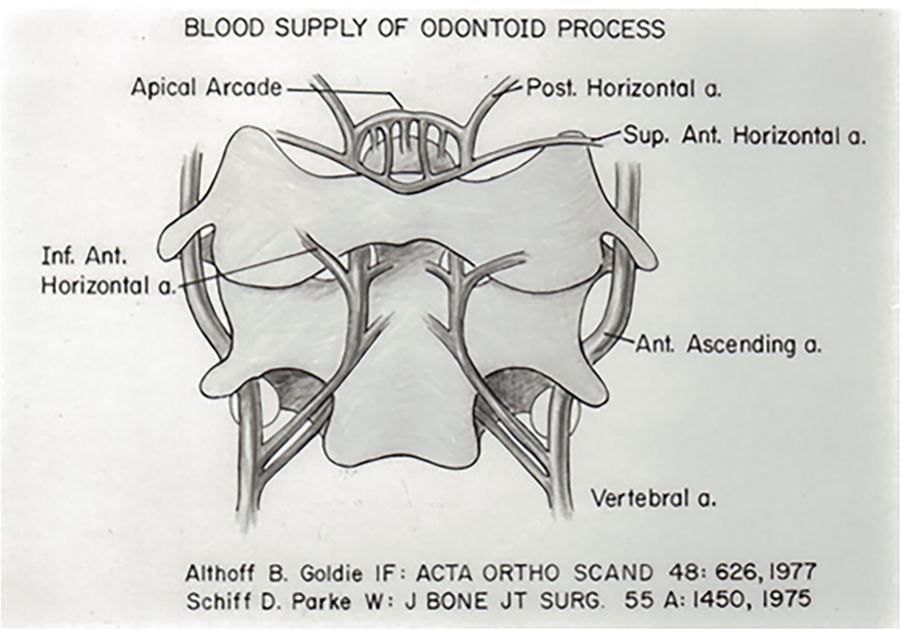
Blood supply of the odontoid process.

The blood supply to the odontoid process originates from two sources (16, 17, 22). The vertebral arteries supply anterior and posterior ascending arteries that course ventral and dorsal to the body of the axis and the odontoid and anastomose in an apical arcade in the region of the alar ligament (Figure 7). These vessels supply the small perforating branches of the body of the axis and the odontoid process. In addition, the anterior ascending arteries and the apical arcade receive contributions from the carotid artery through the base of the skull and the alar ligament. Thus, the organization of the blood supply has an embryologic origin (16, 22).

According to proponents of the embryological formation for the os odontoideum, it is purportedly a result of the failure of fusion between the dens and the axis body (23). If this were the case, the gap between the os odontoideum and the axis should be located below the level of the superior axis facets. However, this has never been demonstrated. In addition, in all patients with the presence of an os odontoideum, a neurocentral synchondrosis is almost always visible. However, os odontoideum is associated with numerous conditions such as segmentation failure with Klippel–Feil syndrome, atlas assimilation, Down syndrome, Morquio syndrome, and skeletal dysplasias (Table 2) (9, 11, 15, 16, 24, 26–30). In our series, there were two sets of twins with spondyloepiphyseal dysplasia. In a study conducted at the Children's Hospital of Philadelphia between 1991 and 2004 on 16 cases of os odontoideum, it was found that half of the patients (eight) reported a history of trauma. Among the remaining eight cases, six exhibited associated congenital abnormalities of the cervical spine and three were diagnosed with genetic syndromes (31). It is also possible that the os odontoideum results from the failure of proper caudal migration of the odontoid process during development (25). An alternative hypothesis proposes the failure of segmentation of the dens from the anterior arch of the atlas, resulting in the “jigsaw sign” (32). Analysis of our database comprising 260 patients revealed that 94 had associated congenital bony abnormalities (36%), with 73 exhibiting syndromic abnormalities and 21 showing Chiari I malformation. The congenital variety showed early presentation as per our population-based study. In addition, it is important to recognize that in congenital syndromic abnormalities, the complication rate, as described in the literature, was 40%, in contrast to 15% in non-syndromic cases (33, 34). Numerous case reports document the presence of os odontoideum in families, suggesting a hereditary nature, and in twins (10–12, 14, 26, 31, 33, 35–37). In the congenital variety, we have recognized that the anterior arch is not hypertrophied, nor is there atrophy of the posterior arch of the atlas. This could be because of early presentation as opposed to those with traumatic origin.

During development, the odontoid process has an apical component from the proatlas (fourth occipital sclerotome), a contribution from the centrum of C1 to form the body of the odontoid process, and involvement of the C2 sclerotome that forms the base (15, 18). Between each of these components is the upper neurocentral synchondrosis, with the lower forming the lowest synchondrosis, also known as the neurodental synchondrosis. It has been suggested that trauma occurring in early childhood between the upper and lower synchondrosis can lead to either a fracture or stress, affecting the vascular supply to the odontoid process (4, 9, 25). The apex would remain viable, and with contracture of the apical and alar ligaments, this pulls the odontoid segment away from the C2 body, resulting in the formation of os odontoideum. This also explains the hypertrophy of the anterior arch of the atlas and the atrophy of the posterior atlas arch. In our series of 260 patients who underwent surgical treatment, 156 had a history of early childhood trauma and 54 had documentation of a normal odontoid process at the initial injury, for which we were able to obtain these images from the emergency departments around the country where these children were first encountered. In these 54 cases, the subsequent development of os odontoideum was documented, and patients were referred due to neck pain, neurological deficit, and acute worsening with trauma. In none of these post-traumatic groups did we observe bony abnormalities, which seemed to have been reserved for the congenital variety.

It is only recently that patients with initial trauma have undergone CT or MRI scanning at the initial injury.

## Conclusions

The etiology of os odontoideum is multifactorial. There does exist a congenital variety in which associated bony and syndromic abnormalities are present. There is a question of whether the congenital variety represents an ossiculum terminale persistens, implying changes in the proatlas segment of the odontoid origin. These individuals present much earlier than the post-traumatic variety. Analysis of a large database contributes to a better understanding of the etiology.

## Data Availability

The original contributions presented in the study are included in the article/supplementary materials, further inquiries can be directed to Arnold Menezes arnold-menezes@uiowa.edu.
